# The Role of Omega-3 and Omega-6 Polyunsaturated Fatty Acid Supplementation in Human Health

**DOI:** 10.3390/foods14193299

**Published:** 2025-09-23

**Authors:** Diogo Gutierres, Rita Pacheco, Catarina Pinto Reis

**Affiliations:** 1 Faculdade de Farmácia, Universidade de Lisboa, 1649-003 Lisbon, Portugal; diogogutierres123@gmail.com; 2 Centro de Química Estrutural, Institute of Molecular Sciences, Faculdade de Ciências, Universidade de Lisboa, 1749-016 Lisbon, Portugal; rita.pacheco@isel.pt; 3 Departamento de Engenharia Química, Instituto Superior de Engenharia de Lisboa, 1959-007 Lisbon, Portugal; 4 Institute of Medicines (iMed. Ulisboa), Faculdade de Farmácia, Universidade de Lisboa, 1649-003 Lisbon, Portugal; 5 Instituto de Biofísica e Engenharia Biomédica (IBEB), Faculdade de Ciências, Universidade de Lisboa, 1749-016 Lisbon, Portugal

**Keywords:** omega-3, omega-6, health benefits, polyunsaturated fatty acids, food supplements

## Abstract

The concept “we are what we eat” is gaining increasing relevance as diet-related diseases and comorbidities continue to rise, while consumers place greater emphasis on healthy lifestyles and acknowledge the pivotal role of nutrition in disease prevention. Among dietary components, omega-3 (ω-3) and omega-6 (ω-6) polyunsaturated fatty acids stand out for their broad spectrum of health benefits. This review explores their potential roles in reducing triglyceride levels, delaying the onset of neurodegenerative disorders such as Alzheimer’s and Parkinson’s diseases, preventing depression, supporting infant brain development, modulating inflammatory processes, and contributing to cancer prevention. The mechanisms of action of these fatty acids are discussed, along with their potential adverse effects—particularly the risk of interactions with anticoagulant medications, which require cautious use. While ω-3 fatty acids are widely recognized for their anti-inflammatory properties, ω-6 fatty acids exhibit both pro- and anti-inflammatory effects, highlighting the importance of achieving a balanced intake. The recommended ω-6:ω-3 ratio, ideally between 4:1 and 1:1, is emphasized as a key element in promoting informed dietary choices. This review also discusses current legislation framework on food supplements, with a focus on challenges such as the lack of stringent regulation regarding supplement content. These gaps underline the need for improved nutritional literacy and stronger regulatory oversight. Ultimately, this review emphasizes the imperative for evidence-based dietary fat recommendations, integrative public health education strategies, the revision and standardization of nutritional guidelines, and the enforcement of robust regulatory frameworks and quality-control protocols across the food supplement industry.

## 1. Introduction

The concept “We Are What We Eat” has evolved into a powerful framework for understanding the intricate connections between human health, dietary patterns, and the wider sociocultural and environmental context. Nutrition influences growth, development, disease prevention, and overall well-being, with macronutrients (carbohydrates, proteins, and fats) and micronutrients (vitamins and minerals) each contributing in distinct yet interconnected ways. Among the various nutritional components, polyunsaturated fatty acids (PUFAs), particularly omega-3 (ω-3) and omega-6 (ω-6), have attracted significant attention for their critical roles in human physiology and their potential therapeutic applications. ω-3 and ω-6 PUFAs are widely recognized for their roles in supporting brain function and cardiovascular (CV) health; these PUFAs are among the most commonly used nutritional supplements, with numerous studies validating their efficacy [1,2]. Notably, their physiological importance is reinforced by the fact that mammals are unable to synthesize these fatty acids (FA) endogenously, making their regular dietary intake essential for maintaining health and preventing diseases.

Although much is known about ω-3 and ω-6 fatty acids, the existing scientific literature tends to focus either on specific health benefits or provide broad overviews of their roles in human physiology, creating gaps in integrating nutritional, molecular, and therapeutic insights. Therefore, this review aims to provide a more comprehensive and detailed overview of the current knowledge on ω-3 and ω-6 PUFAs, covering their nutritional relevance, molecular structures, primary dietary sources, and established and emerging health benefits, as well as other critical aspects associated with their biological functions and therapeutic potential.

Despite their popularity, nutritional supplements such as food-based ω-3 and ω-6 are subject to quality, safety, and efficacy requirements that differ significantly from those applied to medicines, being regulated by different entities. The regulation of nutritional supplements, including those containing ω-3 and ω-6 polyunsaturated fatty acids, is supported by both regional and global frameworks. In Europe, the European Food Safety Authority (EFSA) plays a central role by providing scientific opinions that guide risk assessment and support the development of harmonized legislation across member states. At the global level, the Codex Alimentarius, established by the Food and Agriculture Organization (FAO) and the World Health Organization (WHO), develops international food standards and guidelines, including those relevant to dietary supplements, with the aim of protecting consumer health and ensuring fair practices in the food trade. The inclusion of these organizations is fundamental to contextualizing supplementation practices beyond the national level, as their recommendations provide a reference framework that contributes to consistency in regulatory approaches and facilitates the translation of scientific evidence into public health policies [3,4].

With the growth of the food supplementation market, it becomes increasingly urgent to implement standardized procedures to safeguard consumers. Moreover, the adverse effects and interactions with other substances, such as prescription medicines, are not well known and understood by consumers, posing additional risks.

Gathering information about the biological effects of the ω-3 and ω-6 and the current state of knowledge on existing supplements and their claimed outcomes, this review aims to spread knowledge about their potential and awareness on the importance of having standardized procedures to ensure the safety, quality, and efficacy of these food supplements.

The information presented can be useful for key participants across the supply chain, from manufacturers, who bear responsibility for transparent communication and accurate product information, to pharmacists and vendors, who must be adequately informed to advice and provide appropriate guidance ensuring conscious and safe consumption, and ultimately for consumers, whose health outcomes depend on the reliability of such products.

To guide the analysis, several key questions frame this review: “How much ω-3 and ω-6 should be consumed?”, “Is supplementation necessary or can diet provide the amounts needed?”, “What are their mechanisms of action?”, “What are the main health benefits of omegas?”, “Are there any recently discovered health benefits associated with omegas?”, “What risks and side effects are associated with misuse and how can they be prevented?”, “Are there any interactions with prescribed medicines? If so, which ones?”. Accordingly, this review addresses the effects of ω-3 and ω-6 PUFAs on hypertriglyceridemia, CV health, on brain function and neurodegenerative diseases, on the inflammation process, and on cancer prevention, as well as discussing the main reported adverse effects.

The main databases used for this review were Pubmed and Pubchem. The scientific articles used were all in English, dated between 2010 and 2025, and the keywords used for the search were: “Omega”, “Omega 3”, “ω-3”, “Omega 6”, “ω-6”, “Omega fatty acids”, “Health benefits”, “Cardiovascular”, “Neurodegenerative”, “brain function”, “Depression”, “Depressive disorder”, “Alzheimer’s disease”, “Parkinson’s disease”, “Inflammation”, “anti- inflammatory”, “cancer”. When searched with only “omega 3” as a keyword, there were 27,197 articles available; with “omega 6”, there were 25,301. To further enhance the search, a combination of different keywords was used. Conference proceedings and data from languages other than English were excluded.

## 2. Nutritional Aspects of Diet

### 2.1. Macronutrients and Micronutrients

Macronutrients, such as fats, proteins, and carbohydrates, are required by the body in larger quantities, whereas micronutrients are needed in smaller amounts, such as vitamins and minerals. Generally, the diet can provide both macronutrients and micronutrients at the same time; for example, fish and plant oils, seeds and nuts are the main dietary sources of ω-3 and ω-6 fatty acids (FA). In addition, these same foods also contribute valuable micronutrients, such as vitamins A and D from fish oils, some minerals like zinc, iron, and manganese from plant oils, seeds, and nuts [5,6]. However, despite the variety of dietary sources, deficiencies in certain macronutrients or micronutrients can still occur due to inadequate intake, disease, and other conditions, leading to deficiencies or increased physiological demands. In such cases, supplementation becomes an important strategy to help meet nutritional requirements and prevent associated health problems.

### 2.2. Dietary Patterns and Its Impact

The diet plays a significant role in both physical and physiological health. Having a balanced diet throughout life improves one’s health and helps the body and brain to better develop. Having a balanced diet with all the macronutrients and micronutrients in good quantities is essential. Omega FA, for example, plays a big role in the development of brain function and in the prevention of cardiovascular incidents; others play a role in maintaining the good functioning of other parts of the body, such as the immune system or the metabolism, for example [2,7].

Throughout history, the diet patterns have always been connected to the culture; for example, Portugal, a country situated close to the sea where the consumption of fish and seafood reaches over 50 kg per capita per year [8]. This fact has an impact on the population’s diet and on what nutrients they ingest throughout life. The financial situation of a country also affects the way people eat. In richer countries, people tend to overeat more, which leads to a more overweight population, whereas in poorer countries, this does not happen as much [9,10].

Nowadays, with the industrialization of the food industry, processed foods and bad diet habits are more common than ever, playing a big role in the development of chronic diseases, such as diabetes, high cholesterol levels, and obesity, and increasing the risk of cardiovascular and heart diseases. Besides that, social media is more present than ever, leading to people comparing themselves with what they see online. This exerts considerable pressure to conform to an idealized body image, shifting the focus toward appearance-based eating rather than health-oriented nutrition. Consequently, this might lead to more stress, anxiety, and depression, especially in the younger population. Having a healthy and nutritious diet not only contributes to a healthier body, but also to a healthier mind [2,7,9,10].

### 2.3. Dietary Guidelines and Nutritional Recommendations

Throughout life, nutritional needs in terms of energy and nutrients vary according to age, physiological status, and lifestyle. For example, in infancy and early childhood, optimal nutrition is crucial for healthy growth and cognitive development. The WHO recommends exclusive breastfeeding for the first 6 months of life, followed by continued breastfeeding alongside the introduction of adequate, safe, and nutrient-dense complementary foods [7].

During adolescence, nutritional requirements increase to support rapid growth and development, with particular emphasis on protein, calcium, iron, and overall energy intake. In adulthood, a healthy diet must include fruits, vegetables, legumes (e.g., lentils and beans), nuts, and whole grains (e.g., unprocessed maize, oats, wheat, and brown rice). Adults should consume at least 400 g of fruits and vegetables daily, excluding potatoes, sweet potatoes, cassava, and other starchy roots. The intake of free sugars should be equivalent to less than 10% (ideally 5%) of the total energy intake, which, for an average healthy adult who consumes 2000 calories a day, is equivalent to 50 g of free sugars. Free sugars include added sugars, plus those naturally present in honey, syrups, and unsweetened fruit and vegetable juice. Total fat intake should be less than 30% of total energy, with a preference for unsaturated fats (found in fish, avocado, nuts, and olive oil, for example), such as the ω-3 and ω-6, over saturated fats (found in fatty meat, butter, palm, and coconut oil, for example). This recommendation is based on their effects on blood lipids, as unsaturated fats help regulate blood cholesterol by lowering LDL-C and supporting HDL-C, thereby reducing cardiovascular disease (CVD) risk, whereas saturated fats increase LDL-C, promoting a higher CVD risk. Saturated fats should represent a maximum intake of 10% of total energy, and trans fats (found in baked and fried foods and pre-packaged snacks and foods, for example) that should have a maximum intake of 1% of total energy; salt consumption should be less than 5 g per day. In older age, attention to adequate protein, calcium, vitamin D, and fiber intake becomes particularly relevant in order to maintain muscle mass, bone health, and overall functionality [7].

In order to achieve and maintain a healthy diet, WHO also provides some practical recommendations, such as to include a portion of vegetables in every meal, eat fruits and vegetables raw as a snack, replacing saturated and trans fats with unsaturated fats, steam and boil instead of frying, using healthier oils to cook, such as soybean and sunflower oils, not having salt or high sodium sauces on the table, limiting the consumption of salty snacks, checking the quantity of salt when buying groceries, and limiting the consumption of foods and drinks with high amounts of sugars [7].

## 3. Structure and Omega Fatty Acids Overview

ω-3 and ω-6 are a class of FA. FA can be monounsaturated (MUFAs) or PUFAs, the difference being the number of double bonds in the carbon chain, where MUFAs have only one double bond and PUFAs have more than one. Between ω-3 and ω-6, the difference resides on the placement of the first double bond. ω-3 is an FA with a double bond between the third and fourth carbon atoms from the methyl end of the carbon chain, and ω-6 is the one with a double bond between the sixth and seventh carbon atom from the methyl end of the carbon chain. The omegas can be further divided into four different groups, depending on the size of the carbon chain. If it has a carbon chain of one to six carbon atoms, it is a Short-Chain Fatty Acid (SCFA), also known as Volatile Fatty Acid (VFA) [11]; if it has seven to 12 carbon atoms, it is a Medium-Chain Fatty Acid (MCFA) [11]; if it has 14 to 18 carbon atoms, it is a Long-Chain Fatty Acid (LCFA) [12]; and if it has more than 20 carbon atoms, it is a Very Long-Chain Fatty Acid (VLCFA) [13].

The main ω-3 PUFAs being focused on this paper are ɑ-linoleic acid (C_18_H_30_O_2_) (ALA), eicosapentaenoic acid (C_20_H_30_O_2_) (EPA), and docosahexaenoic acid (C_22_H_32_O_2_) (DHA), and the main ω-6 PUFAs are linoleic acid (C_18_H_32_O_2_) (LA), the predecessor of arachidonic acid (C_20_H_32_O_2_) (ARA) [12,14].

ALA and LA are classified as essential FA because they can only be acquired from dietary sources and cannot be synthesized endogenously. Even though the other PUFAs (EPA, DHA, and ARA) can be synthesized endogenously from ALA and LA, the conversion efficiency is very low, especially for DHA from ALA in humans (typically <5% for DHA and 5–10% for EPA). Therefore, dietary intake of EPA, DHA, and ARA is often necessary to meet physiological needs, particularly for neural, cardiovascular, and immune functions. The main dietary sources of the omegas are, for example, fish (e.g., salmon, tuna, or sardines), nuts (e.g., nuts or cashew) and seeds (e.g., chia seeds or flax seeds) [12,15,16,17,18].

The ω-3 FA is involved in the development of brain function and preventing neurodegenerative diseases, such as Alzheimer’s and dementia and, in some studies, in preventing cancer [19,20]. ω-3 and ω-6 also have an impact on blood cholesterol levels, helping to prevent cardiovascular problems and on the inflammatory process, helping the body give a more adequate response when needed [2,12,17,19].

For the omegas to have the expected health benefits, the ratio between the ingestion of ω-6 and ω-3 is key. Nowadays, many diets have a bigger intake of ω-6 compared to a much lower intake of ω-3. A ratio of 4:1 to 1:1 of ω-6 to ω-3 is considered the most balanced and to have health benefits. A variation on this ratio can be more effective for different diseases; for example, a ratio of 4:1 was associated with a bigger prevention of cardiovascular events, whereas a 2.5:1 ratio reduced rectal cell proliferation in patients with colorectal cancer [12,21].

## 4. Main Dietary Sources of ω-3 and ω-6

As mentioned before, ω-3 and ω-6, more specifically, ALA and LA, are considered essential FAs because humans cannot produce them and, even though the other omegas that are being mentioned in this review can be synthesized through metabolism pathways in the human organism starting from ALA and LA, their production is limited because they use the same elongases and desaturases enzymes in the metabolic pathways. With that in mind, it is necessary to have good and healthy amounts of all omegas from the diet.

A study using data from the National Health and Nutrition Examination Survey from 2003 to 2008, based on the American population, concluded that the population consumed, from food sources, an average of 0.17 g/day of ω-3 PUFAs; more specifically, EPA and DHA, when the American Heart Association recommends consuming 0.5 g/day [22]. Nowadays, the National Academy of Medicine recommends a daily intake of 1.6 g/day and 1.1 g/day of ALA for men and women, respectively [23]. From the survey, it was possible to determine that fish is the main source of EPA and DHA in the population since the ones that consumed a deficient amount of ω-3 were the ones that consumed less fish in their diet, contrary to the population with the highest intake where fish was the main ω-3 source [22]. One reason for fish to be such a great source of EPA and DHA is the fact that they consume algae. Algae is a great source of EPA and DHA [24].

ω-3 PUFAs can be found in some fish, nuts, seeds, and plant oils; more specifically, EPA can be found in great amounts in herring, wild sardine, and pollock roe, although its presence is rather rare in plants; DHA can be found in flying fish, herring, pollock, salmon roe, and jackal berry; ALA, on the other hand, is more common in plants and seeds than in fish, being found in flaxseed, chia, and canola oil. These are not all the sources of ω-3 but are some of the more noteworthy ones [6,12,16,17].

One important point to consider when trying to consume enough ω-3 is that, since fish are the main source of EPA and DHA, as previously mentioned, frequent consumption of certain fish, more specifically, the prime hierarchy predators like tuna, can expose the human body to high levels of methylmercury, found in these species because of their environment. Methylmercury has a neurotoxic effect, making it very important to have alternate sources of these nutrients in the daily diet. This problem can spread to food supplements based on extracts of fish oils, where methylmercury can also be present [12]. ω-6 PUFAs can be found in many oils and seeds, such as corn sunflower, soybean, canola, safflower and flaxseed oils, chia, walnuts, hazelnuts, and almonds. Smaller amounts of LA can also be found in some vegetables and fish, and ARA can be found mostly in meat, eggs, and dairy products, but also in some fish, like salmon, herring, sardine, trout, and cod, but in smaller amounts [15,25,26,27]. A more detailed list of some of the food sources and the amount of each omega fatty acid they contain can be found in Table 1.

## 5. Metabolism and Bioavailability of ω-3 and ω-6

**Figure 1:**
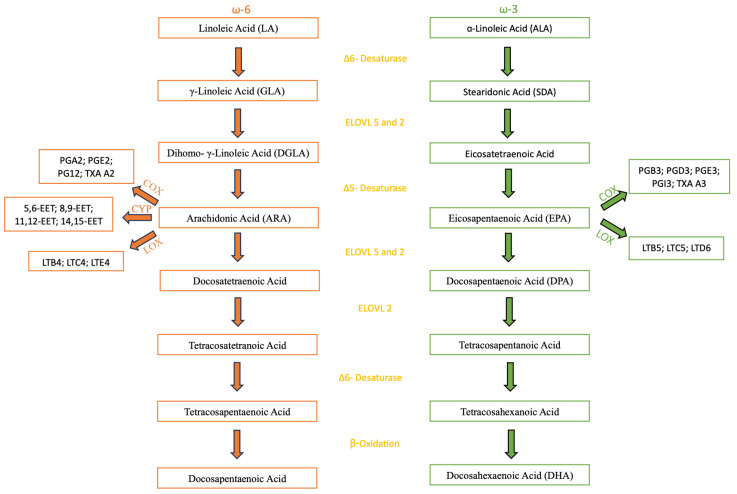
Metabolic pathways of Omega-6 and Omega-3 Fatty Acids. Adapted from [26]. ω-6: Omega-6; ω-3: Omega-3; LA: Linoleic Acid; ALA: ɑ-Linoleic Acid; GLA: γ-Linoleic Acid; SDA: Steridonic Acid; DGLA: Dihomo-γ-Linoleic Acid; ARA: Arachidonic Acid; EPA: Eicosapentaenoic Acid; DPA: Docosapentaenoic Acid; DHA: Docosahexaenoic Acid; ELOVL: Elongase; COX: Cyclooxygenase; LOX: Lipoxygenase; CYP: Cytochrome P450; PG: Prostaglandin; TXA: Thromboxane; EET: Eicosatrienoic Acid; LT: Leukotrienes.

In plants and animal-based food, ω-3 and ω-6 are mostly found in the form of triacylglycerols (TAG), phospholipids (PL), Diacylglycerols (DAG), and Cholesterol Esters (CL) [26], but they may also appear as free fatty acids (FFA) and ethyl esters (EE) [19], with PLs being the most bioavailable because of their aliphatic characteristics. When ingested, these PUFAs are mostly assimilated into TAG and their digestion starts in the stomach, where gastric lipases break down TAGs into DAG and FFA. The complete digestion occurs then in the intestine lumen with the aid of bile salt and pancreatic lipases. ω-3 FAs in the form of EEs, TAGs, or PLs have shown to be better digested and absorbed when the fat content of the food is high because it enhances the activity of the pancreatic enzymes [19]. The release of the FAs allows absorption and transportation into the bloodstream. After that, the FAs can either undergo esterification into cellular lipids as PL, TAG, or CE; a ꞵ-oxidation to provide energy; or go through the metabolic pathway described further, and illustrated on Figure 1, to form the other ω-3 and ω-6 FAs [26]. The latter occurs mostly in the liver, occurring in other tissues but in an insignificant amount [27]. As for the biosynthesis in the human body, both ω-3 and ω-6 metabolism share the same enzymes through their metabolic pathways, a group of elongases and desaturases situated mostly in the liver, starting from ALA and LA, respectively. The first step in the metabolic pathway is catalyzed by the Δ6-fatty acid desaturase (FADS) that adds a double bond at the sixth carbon position from the carboxyl group of ALA and LA, resulting in the formation of Stearidonic Acid (SDA) and γ-Linoleic Acid (GLA), respectively. This first step is of special importance in the metabolic pathway because the Δ6-FADS is a rate-limiting step in humans, meaning it is the slowest one, therefore dictating the overall reaction rate. The second step is an elongation that leads to the synthesis of eicosatetraenoic Acid (ETA) and Dihomo-eta-Linoleic Acid (DGLA). After that, Δ5-FADS adds a new double bond to the fifth carbon from the carboxyl group, resulting in the formation of ARA and EPA. From there, both metabolic pathways still undergo some more steps, the most important being in the ω-3 pathway, which ends up in the formation of DHA, one of the PUFAs being studied in this review. Both metabolic pathways are represented on Figure 1 [26].

The highest rate of conversion from ALA to EPA and DHA in the hepatic cells occurs when the ratio of ALA and LA is 1:1. The conversion rates recorded were 17% and 0.7% for EPA and DHA, respectively. These data highlight the importance of having DHA supplementation or, at least, being incorporated into the daily diet food containing DHA because the conversion rate from ALA is low [18]. As mentioned, the ω-3 and ω-6 PUFAs share the same enzymes through their metabolic pathways and, because of that, the rate of conversion of ALA and LA into EPA and ARA, respectively, depends on the availability of the substrate. Nowadays, many diets have much higher amounts of ω-6 than ω-3. It is expected that LA, being the dominant substrate, becomes more converted into ARA than ALA into EPA and DHA, thus explaining why, usually, the levels of ω-6 PUFAs are higher in the organism. This could explain the observed low rates of conversion of ALA into EPA and DHA [2]. A report made by the Food and Agriculture Organization (FAO) pointed to the fact that conversion rates of both ω-3 and ω-6 can also be affected by other factors. The Δ5-FADS appears to have a lower rate of activity in people that are diabetic, and the Δ6-FADS also appears to have a lower rate of activity when insulin levels are low or there is a deficiency of proteins and some vitamins like iron, zinc, copper, and magnesium. Lower rate of activity of these enzymes results in lower rates of conversion of ALA and LA into EPA and ARA, respectively, since both enzymes have a present role in the metabolic pathways. This, once again, reflects on the importance of having implemented supplementation of these nutrients into the diet to get the health benefits from them [31].

When needed, ω-3 and ω-6 FAs can be used to form different types of oxylipins by oxygenase enzymes, such as lipoxins, resolvins, protectins, and maresins. Oxylipins are bioactive signaling molecules with different functions such as pro/anti-inflammatory and immune response regulation. These reactions are led by specific enzymes, cyclooxygenase (COX), lipoxygenase (LOX), and cytochrome P450 (CYP) [32].

ω-3 FA concentration can be measured in plasma, serum, blood cells, and lymph. When trying to evaluate the content of FAs in the diet in the medium–long term, plasma is the best indicator; when evaluating a long-term supply, blood cells are the best indicator. There are, also, other ways to calculate the bioavailability of ω-3, the most important being the ω-3 index and maximum concentration (Cmax). The ω-3 index is the proportion of the sum of EPA and DHA content in the total FA content in the erythrocyte membrane, expressed as a percentage. This is a good indicator of the bioavailability for the past 80–120 days due to the long lifetime and the abundance of these cells. The Cmax of EPA and DHA in the plasma can be determined 5 to 9 h after administration, while the persistent levels can be achieved after 2 weeks of daily supplementation [12].

ω-3 FAs are present in many tissues and organs, such as the heart, nervous tissue, and the retina. It is also present in the cerebrospinal fluid. A way to improve its passage through the blood–brain barrier (BBB) up to 10 times is by using a carrier particle. In the case of DHA, the carrier particle used is 1-lyso, 2-docosahexaenoyl-glycerophosphocholine (LysoPC-DHA). It is a brain-specific particle that improves the passage through the BBB but not to other tissues or organs [12].

## 6. Main Health Benefits of ω-3 and ω-6

### 6.1. Effects on Hypertriglyceridemia

**Figure 2:**
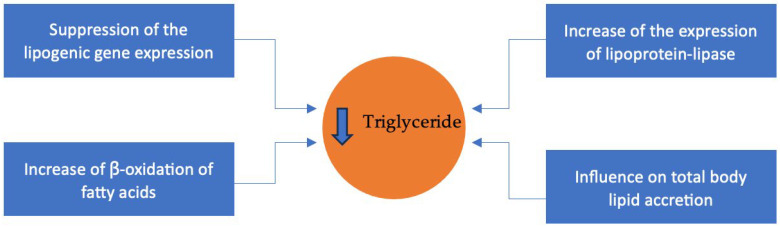
Molecular Mechanisms of Triglyceride Lowering by Omega-3 Fatty Acids.

The mechanism behind the effect on hypertriglyceridemia is still not fully understood, but studies point towards it being due to the suppression of the lipogenic gene expression, the increase of ꞵ-oxidation of FA, the increase of the expression of lipoprotein-lipase (LPL), and the influence on total body lipid accretion [33]. Figure 2 illustrates the four mechanisms by which ω-3 fatty acids reduce triglyceride levels.

The suppression of lipogenic gene expression is achieved by decreasing the expression of sterol regulatory element-binding protein 1c (SREBP). SREBPs are a membrane-bound enzyme that, when cleaved, transcribe enzymes that are involved in the production of cholesterol, LDL, and FAs. A diet rich in ω-3 PUFAs decreases the activation of the SREBP 1c due to negative feedback, leading to a decrease in the production of cholesterol through the enzymes that it regulates (farnesyl diphosphate synthase and 3-hydroxy-3-methylglutaryl-CoA reductase) [33].

To produce energy from FAs, the body uses ꞵ-oxidation. ω-3 PUFAs can enhance the rate of ꞵ-oxidation, primarily through their regulatory effects on carnitine acyltransferase (CAT1) and acetyl-CoA carboxylase (ACC). CAT1 acts on facilitating the transportation of FAs into the mitochondria. Inside it, the FAs are converted into acyl-CoA, which serves as a precursor to acetyl-CoA, a central molecule in ATP production through various metabolic pathways. Additionally, EPA indirectly stimulates ꞵ-oxidation by inhibiting ACC, the enzyme responsible for synthesizing malonyl-CoA, a potent inhibitor of CAT1. Furthermore, ω-3 PUFAs have been shown to reduce CAT1’s sensitivity to malonyl-CoA, further promoting FA utilization for energy production [33].

LPL is an enzyme responsible for the removal of triacylglycerol components from chylomicrons, LDL, and very-low-density lipoproteins (VLDL) in the blood. ω-3 PUFAs have been shown to increase the activity of LPL, reducing the levels of LDL, VLDL, and chylomicron size [33].

ω-3 PUFAs can also influence total body lipid accretion. Studies have demonstrated that the use of ω-3 PUFAs for more than 6 weeks might increase the body’s metabolic rate and decrease total body fat. Participants in the studies showed an increase in lean body mass, a reduction in adipose tissue, elevated resting metabolic rate, greater energy expenditure during physical activity, and enhanced ꞵ-oxidation, both while resting and during physical activity [34,35]. These facts occur because ω-3 PUFAs can act as a ligand for peroxisome proliferator-activated receptors (PPARs). PPARs can then regulate both ꞵ-oxidation and glucose metabolism and change the basal metabolism of the cell [33].

Through all these four mechanisms, ω-3 PUFAs support their efficacy in the reduction of the level of triglycerides. Therefore, daily intake of ω-3 PUFAs, either through the diet or with supplementation, might be advisable for patients with diagnosed hypertriglyceridemia and even healthy people as a way of prevention.

### 6.2. Effects on Cardiovascular Health

Nowadays, the effects of ω-3 in cardiovascular health are still a subject of debate. Throughout the many trials and the meta-analysis available on the subject, the opinions are not unanimous, and while the cardioprotective effects of the ω-3 are noticeable in most studies, others do not show the same results.

One of the first landmark trials for ω-3 supplementation was the GISSI-P, a study with approximately 11,000 subjects and almost 4 years of follow-up that showed that a supplementation of 1 g/day of ω-3 (EPA + DHA) decreased the risk of death, non-fatal acute myocardial infarction (MI), and stroke in patients with recent MI (<3 months). However, many subsequent studies did not achieve the same results [36].

More recently, in 2018, three large trials were released, but also with divergent conclusions. The trials were called ASCEND, VITAL, and REDUCE-IT. The ASCEND trial included 15,480 patients and had a follow-up period of 7.4 years. It used 1 g/day of EPA + DHA supplementation as the primary prevention in patients with diabetes but found no reduction in CVD risk. Through the study, an 18% relative risk reduction in vascular death, defined as death from coronary heart disease (CHD), stroke, or other vascular causes, was found [37].

The VITAL trial included 25,871 subjects and had a median follow-up of 5.3 years. It used 2000 IU/day of Vitamin D and 1 g/day of EPA + DHA supplementation for the primary prevention of CVD and cancer. Like the ASCEND trial, the VITAL trial found no significant difference between the intervention and the placebo groups in risk reduction but found a significant reduction in total MI. It also showed that patients who consumed less than 1.5 fish meals per week and then had ω-3 supplementation had a significant reduction in major adverse cardiovascular events (MACE) and risk of MI by 19% and 40%, respectively, which points to the importance of having a threshold level of ω-3 to have the therapeutic effects [38].

The REDUCE-IT trial included 8179 patients with established CVD or diabetes receiving statin therapy. The patients were followed for a median of 4.9 years. It used 4 g/day of icosapent ethyl, a highly purified EPA formulation. The primary endpoint of this trial was CVD death, non-fatal stroke, CV revascularization, and unstable angina, and a reduction of 25% was observed in the treatment group, as well as a reduction of 26% on the secondary endpoint (MACE). In the USA population, there was a significant relative risk reduction of 30% and a 2.6% absolute risk reduction in all-cause mortality. While the other populations included in the trial also showed significant reductions in the primary and key secondary endpoints, it was more noticeable in the USA [39].

In the last years, there were some noteworthy meta-analyses published about this topic, which, like the previously mentioned trials, highlighted inconsistent results. One in particular is the meta-analysis by Bernasconi et al. [40], where ω-3 supplementation was associated with a risk reduction of MI (relative risk (RR) 0.87), CHD events (RR 0.90), fatal MI (RR 0.65), CHD mortality (RR 0.91), and CVD events (RR 0.95), although the latter was not statistically significant. It is important to mention that the risk reduction of MI and CVD events seems to be dose-dependent. This meta-analysis included a total of 40 studies and examined not only the effects on the risk reduction of various outcomes but also the level of heterogeneity for each result; this information is summarized in Table 2 [40].

Overall, this meta-analysis concluded that ω-3 supplementation, despite having some trials that do not show results in reducing risk in many outcomes, most of the studies support that ω-3 supplementation is effective, as previously discussed [40].

**Figure 3:**
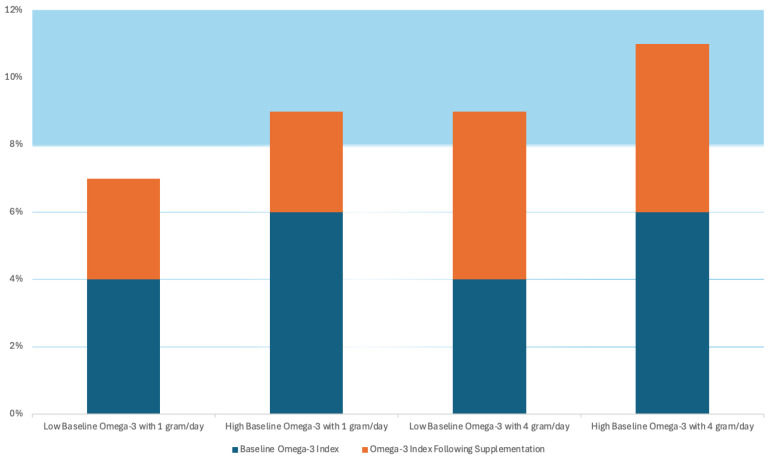
Hypothetical ω-3 supplementation dose and threshold effect according to baseline ω-3 levels. Adapted from [36].

As mentioned, in certain outcomes (CVD events and MI), the benefits of the ω-3 appear to be dose-dependent, which can explain why, in studies like GISSI-P, where the subjects were from Italy, a country where fish is very much present in the daily diet, there is presence of a threshold level of ω-3 that contributes to it reaching a therapeutic level of ω-3 with lower dose supplementation (1 g/day), where other studies showed better results with higher dose supplementation (4 g/day). This idea is well demonstrated in a hypothetical representation present in Figure 3 [36]. In this figure, the blue columns represent the baseline levels of ω-3, and the orange columns represent the supplementation levels. The study suggests that an ω-3 index > 8% is necessary to predict a lower CV risk, and it can also, potentially, reduce the risk of fatal CHD by approximately 35% [36].

The effect of ω-3 has also been studied in heart failure (HF). GISSI-HF was a trial that included nearly 7000 HF patients (91% with reduced ejection fraction (HFrEF)) with a median follow-up of 3.9 years. It used 1 g/day of ω-3 supplementation against placebo and found a number needed to treat (NNT) of 56 to prevent one death or NNT of 44 to avoid one death or hospital admission for CVD reasons. These results lead the American Heart Association (AHA) to provide a class IIa indication that ω-3 supplementation is reasonable for the treatment of patients with HFrEF [36].

In late 2020, two more studies about the effects of ω-3 supplementation on CV health, OMEMI, and STRENGTH found no CV benefits in high-risk patients. Even though these trials add to the confusion around the benefits of ω-3 supplementation on CV diseases, they did not cancel out the findings of previous trials and meta-analyses that concluded a more positive view on the usefulness of ω-3 supplementation [36].

In terms of the mechanisms that allow ω-3 to have the benefits described, it was suggested to be associated with the potential modulation of key CV risk factors, such as high blood pressure, high serum triglycerides, low high-density lipoprotein (HDL)- cholesterol, elevated post-prandial lipidemia, endothelial dysfunction, cardiac arrhythmia, heart rate, and heart rate variability, as well as a tendency towards thrombosis and inflammation. Many studies have indeed confirmed that EPA and DHA have effects by improving all the above-mentioned traits while increasing both low-density lipoprotein (LDL) and HDL-cholesterol. Throughout the studies, the reduction of inflammation is marked by the reduction of the C-reactive protein (CRP), pro-inflammatory cytokines, tumor necrosis factor (TNF)-α, and interleukin (IL)-6 [41].

While ω-3 has an acknowledged effect on CVD risk reduction, especially EPA and DHA, ω-6 PUFAs do not have such acknowledgement. A study by Chowdhury et al. [42] found no evidence of dietary intake of ω-6 having any effect in reducing CVD risk. On the other hand, a meta-analysis published by Farvid et al. [43] estimated that replacing 5% of the dietary energy of saturated FA with LA was associated with a lower risk of CHD events and CHD deaths by 9% and 13%, respectively [43].

Even though there is much evidence of ω-3 PUFAs having many cardioprotective effects, the evidence on ω-6 PUFAs is not so clear. This lack of conclusive information highlights the need for further investigation to better understand this topic.

### 6.3. Effects on Brain and Central Nervous System

#### 6.3.1. Alzheimer’s and Parkinson’s Disease

Alzheimer’s disease (AD) and Parkinson’s disease (PD) are the two most prevalent neurodegenerative diseases, affecting millions of people over the age of 65 years, and, with the extension of life expectancy, the number of people affected by these diseases tends to grow [44].

The reasons behind the development of AD are still not fully understood, but it is believed to be connected to a combination of factors, like environmental and genetic factors. Nowadays, there are three specific gene mutations that are connected to the development of this disease by affecting the production of amyloid-ꞵ [45]. This disease is characterized by amyloid plaques and protein accumulation in the brain, contributing to loss of connection between nerve cells, consequently leading to brain tissue death [44]. The main symptoms include dementia, memory loss, and cognitive decline [1].

The pathogenesis behind PD, like AD, is still not fully understood but is believed to be connected to environmental and genetic factors. The disease is characterized by the loss of dopaminergic neuronal cells in the substantia nigra pars compacta, a region in the brain responsible for movement control, and the accumulation and aggregation of ɑ-synuclein protein in the form of Lewy bodies. The main symptoms include bradykinesia, resting tremor, muscular rigidity, and postural instability [46].

Although AD and PD have different pathogeneses and clinical manifestations, they share some common mechanisms, like mitochondrial dysfunction, neuroinflammation, and oxidative stress. It is suggested that ω-3 PUFAs, more specifically DHA, are connected to these diseases and can play a role in preventing their development [1].

A higher intake of DHA has been associated with a lower risk of developing cognitive impairment or AD. DHA induces the activation of synaptophysin-1, which is a glycoprotein found in synaptic vesicles that facilitates synaptogenesis and enhances cognitive performance. The biosynthesis and consequent phosphorylation of synaptophysin-1 are regulated by a brain-derived neurotrophic factor. DHA promotes synaptic transmission by upregulating the brain-derived neurotrophic factor, thereby augmenting neural plasticity. DHA can also inhibit tau phosphorylation [1]. Tau is a microtubule-associated protein that, when hyperphosphorylated, can cause microtubule decomposition and the accumulation of neurofibrillary tangles [47]. DHA can also mitigate amyloid-β-caused neurotoxicity, as well as neuronal apoptosis by inhibiting the formation and aggregation of amyloid-ꞵ peptides [1]. Between the studies available on the effects of ω-3 PUFAs on AD, the heterogeneity is significant, having some studies reporting a beneficial effect of the use of ω-3 PUFAs supplementation and others mentioning no observed effect [44]. This highlights the necessity of more and better studies to be made.

Although the mechanisms are not fully understood, DHA is also believed to be capable of slowing and preventing the development of PD. DHA can regulate the expression of ɑ-synuclein and maintain synaptic homeostasis and neuronal activity. It can also increase the expression of glial-derived neurotrophic factors (GDNFs), preventing glial dysfunction [1].

Some studies have tried to establish a correlation between the consumption of ω-3 PUFAs and the development of PD. While many report promising results, suggesting that ω-3 PUFAs may slow disease progression, others fail to demonstrate significant associations. This heterogeneity in findings may be explained by multiple factors, including differences in study design (observational vs. interventional), variability in dietary assessment methods, supplementation dosage and duration, the specific type of ω-3 studied (e.g., EPA vs. DHA), and differences in baseline dietary patterns. Moreover, genetic background, lifestyle factors, and heterogeneity in PD pathology and clinical phenotypes may influence outcomes and contribute to inconsistent findings. Another important consideration is the timing of ω-3 intake in relation to disease onset, as neuroprotective effects may depend on early vs. late exposure. Taken together, these factors highlight why results are mixed and underline the complexity of establishing a causal relationship. Rather than being contradictory, these findings underscore the multifactorial nature of PD and the need for well-designed, long-term, randomized controlled trials to clarify the neuroprotective potential of ω-3 PUFAs.

When it comes to the effects of ω-6 PUFAs, more specifically ARA, the results are very contradictory. While the consumption of ARA is essential for normal brain function, excessive consumption can lead to neuroinflammation and neuronal damage. It is also suggested that a high ratio of ω-6:ω-3 can be linked to the development of neurodegenerative diseases, with a ratio closer to 1:1 being one of the most recommended [44].

Studies on rats have shown that the consumption of ARA can help prevent cognitive impairment associated with the abnormal processing of amyloid precursor protein (APP), therefore preventing the formation of insoluble amyloid-ꞵ in neuritic plaques. On the other hand, other studies on rats have shown that ARA can augment the production and deposition of amyloid-ꞵ. The difference can be due to differences in doses applied, but such is not specified [1].

Eicosatrienoic acid (EET) is a metabolite of ARA that is widely distributed on the brain and shows antioxidant and anti-inflammatory properties, constituting a therapeutic target in PD due to those properties. Studies on the *Drosophila* model show that it can increase the expression of antioxidant enzymes and reduce oxidative stress and inflammation. Moreover, in vitro studies have shown that ARA can promote the ɑ-helical folding of ɑ-synuclein, resulting in reduced neuronal toxicity. In PD, ɑ-synuclein typically aggregates into toxic ꞵ-sheet structures, whereas in healthy neurons, it predominantly exists as non-toxic ɑ-helical oligomers [1].

In short, while a higher intake of ω-3 PUFAs is associated with enhanced synaptic plasticity, inhibition of inflammatory responses, protection of cholinergic neurons, and brain nerve health, ω-6 PUFAs and their metabolites have both pro-inflammatory and anti-inflammatory effects, leaving doubts about their beneficial effects and revealing the need for more research.

#### 6.3.2. Depressive Disorder

Depressive disorders, more commonly known as depression, are a common mental disorder that affects 4% of adult males, 6% of adult females, and 5.7% of the population over 60 years old. Its main symptoms are feeling sad, irritable, empty, and with no interest or pleasure in activities [48].

ω-3 PUFAs have shown promising results as an emerging auxiliary treatment for depression. Preclinical studies on rats fed with a diet rich in EPA for 6 weeks showed increased levels of dopamine and serotonin in the hippocampus. Other studies on offspring rats showed that those lacking ω-3 PUFAs developed nerve damage in the hippocampus and had reduced levels of serotonin and norepinephrine. This points to the capability of ω-3 PUFAs in preventing depression-like behavior. In addition, studies have shown that there is a negative relation between the levels of cortisol in depressed patients [1] (cortisol levels are usually high in depressed patients [49]) and the levels of EPA and DHA in the blood due to its effects on the hypothalamic–pituitary–adrenal (HPA) axis. It has also been shown that patients with depression who increase the intake of EPA for 8 weeks show reduced HPA axis activity and improved depression symptoms [1].

ω-6 PUFAs and their effects on depression are not so clear. Anandamide (AEA) is an endocannabinoid derived from ARA that, in some studies, appears to be inversely related to depression [50], while in other studies, it is said to impair serotonin neurotransmission in the prefrontal cortex (PFC), inducing a depression-like phenotype in mice. Other conflicting studies on PFC samples of postmortem humans previously diagnosed with depression show that DHA levels were reduced and the ratio of ARA:DHA increased, while other studies also using PFC samples of postmortem humans previously diagnosed claim that there were no changes observed [1].

#### 6.3.3. Brain Development of Specific Population

When it comes to the effects of ω-3 on infant brain development, the main PUFA is DHA. While there are studies that report adverse to no effects, most studies report a beneficial effect. For example, a study conducted in Europe between 1992 and 2013 found that supplementation with DHA and ARA improved problem-solving skills at 9 to 10 months and led to fast information processing skills at 6 years of age. Another study, also testing DHA and ARA supplementation, showed a higher sweep visual-evoked potential (VEP) acuity at 12 months, increased mental development index at 18 months, better inhibitory function at 3 to 5 years of age, and better verbal and full-scale IQ at 6 years of age compared with a control group without supplementation [51].

Between the results of the studies, there is a difference that is noteworthy between premature infants and term infants. Starting in the last trimester of gestation until the first 2 years of life, there is an accumulation of DHA from the womb that is lacking in premature infants. This may partly explain why the positive cognitive effects of DHA supplementation are more consistent in studies involving premature infants rather than those involving term infants. This does not fully explain the heterogeneity in results because there are conflicting results even between studies that only involve premature infants [51].

In brief, the usage of DHA in infants might be important, especially in premature infants, and it improves visual acuity, visual recognition memory, attention, psychomotor and mental development, and the maturation of the brain’s white matter [51].

### 6.4. Effects on the Inflammatory Process

Inflammation is a natural process and includes three stages: initiation, development, and extinction. If the last one fails, it becomes chronic inflammation, leading to the possible development of other diseases. The main signs of inflammation are redness, heat, swelling, pain, and loss of function [52]. There are many mediators that participate in inflammation, such as cytokines like interleukin (IL)-1ra, IL-4, IL-6, IL-8, IL-10, IL-11, IL-13, and transforming growth factor (TGF)-ꞵ [32].

ω-3 and ω-6 PUFAs can generate mediators that have a potent influence on the inflammation process through two pathways, non-enzymatic or enzymatic [32].

Since PUFAs have double bonds, they are susceptible to free radicals and prone to free radical-induced autoxidation and photodegradation, leading to the formation of non-enzymatic metabolites, such as phytoprostanes (PhytoPs) from ALA, isoprostanes (IsoPs) from EPA and ARA, and neuroprostanes (NeuroPs) from DHA. From these, PhytoPs and NeuroPs seem to be the ones with effects on the inflammatory process [32]. PhytoPs appear to be able to modulate the synthesis and overall level of PGs during the inflammatory process [53], and NeuroPs have potent anti-inflammatory effects, similar to protectins [32].

When it comes to the enzymatically originated mediators from PUFAs, they are called oxylipins, a broad term that includes many derivatives, including specialized pro-resolvin mediators (SPMs). SPMs are a novel group of compounds that derive from ω-3 and ω-6 PUFAs and include lipoxins, resolvins, protectins, and maresins. ω-3 and ω-6 PUFAs are oxidized by specific groups of enzymes, COX, LOX, and CYP. Generally, oxylipins originating from ω-3 PUFAs have more of an anti-inflammatory effect, while ω-6 PUFAs originating from oxylipins have more of a pro-inflammatory effect. The diet and supplementation influence the amount of each FA present in the organism; therefore, the amount of oxylipins originated from each PUFA. Cytosolic phospholipase A2 (cPLA2) is the enzyme responsible for releasing the PUFAs from the sn-2 position of phospholipids, making them available to form oxylipins [32].

Oxylipins can also be classified based on their precursors. Oxylipins derived from LA and ALA are called octadecanoids, from ARA and EPA are eicosanoids, and from DHA are docosanoids [32].

Besides the mediators synthesized from ω-3 PUFAs, another factor that contributes to the anti-inflammatory effects of EPA and DHA is the fact that they compete with ω-6 PUFAs’ enzymes to produce oxylipins. As mentioned, ω-3 PUFAs originate oxylipins with anti-inflammatory effects, and ω-6 PUFAs originate oxylipins with pro-inflammatory effects since they compete for the same enzymes to produce those oxylipins. A higher concentration of EPA and DHA means that the enzymes are less available for ω-6 PUFAs. Besides that, ω-3 PUFAs are also linked to the decrease of the levels of various inflammatory markers, such as cytokines like IL-1, acute-phase proteins like CRP, and adhesion molecules [2].

Since, as mentioned, ω-3 and ω-6 PUFAs have both anti- and pro-inflammatory effects, respectively, the concept of ω-6:ω-3 ratio becomes important again. A higher ratio might increase the levels of pro-inflammatory oxylipins and contribute to the resolution of the inflammatory process. On the other hand, a higher intake of ω-3 PUFAs can lower the amounts of ω-6-derived oxylipins and promote the correct initiation of the inflammatory process. A balanced ratio of 1:1 appears to be the most advisable [44].

### 6.5. Effects on Cancer

Cancer is a complex disease characterized by the uncontrolled growth and division of abnormal cells. These cells can form a mass known as a tumor, which may be benign (non-cancerous) or malignant (cancerous). Malignant tumors have the potential to invade nearby tissues and spread to other parts of the body through the bloodstream or lymphatic system in a process known as metastasis. This ability to spread makes cancer particularly challenging to treat [54].

There are studies that show an association between the intake of ω-3 PUFAs and a reduced risk of many types of cancer, such as leukemia [55], breast cancer [56], colon cancer [57,58], prostate cancer [59], and melanoma [60].

On the other hand, ω-6 PUFAs, more specifically ARA, are associated with cancer-causing effects. Inflammation is a well-known factor in cancer development, and, as mentioned in the point above, ω-6 PUFAs are more generally known to have a pro-inflammatory effect, being associated with cancer development through the production of pro-inflammatory eicosanoids, such as PGE2, which enhances tumor cell proliferation and angiogenesis. However, it is also believed that ω-6 PUFAs can inhibit the growth and development of cancer through a mechanism that is also associated with ω-3 PUFAs, that is, the induction of reactive oxygen species (ROS) production and mitochondrial damage, causing the death of the cancer cells [1].

The ω-6:ω-3 PUFAs ratio is also a factor that can hypothetically influence the development of cancer. Lower ratios are associated with the upregulation of the tumor suppressor SMAR1 expression and downregulation of the oncogenic factor MARBP Cux/CDP expression. The increased SMAR1 expression also stimulates the expression of the p21 protein, inhibiting cell cycle progression [1].

Besides the reduction of inflammation, ω-3 PUFAs, more specifically EPA and DHA, also have other known mechanisms through which they might influence cancer development. DHA has shown ability to induce apoptosis in human MCF-7 breast cancer cells through caspase-8 activation [61]. EPA and DHA have also been shown to significantly increase the mitochondrial membrane potential. This increased potential leads to the increase in ROS production in colorectal cancer cells, stimulating a proapoptotic mechanism by changing the active form of the caspase-3 and caspase-9 expression [1].

The production of nitric oxide (NO) occurs in the mitochondria and is catalyzed by mitochondrial nitric oxide synthase (mtNOS). Inducible nitric oxide synthase (iNOS) is an enzyme that produces NO. iNOS and NO are associated with cancer development. Peroxidation products of EPA and DHA can inhibit iNOS induction, consequently reducing NO production in hepatocytes. An anti-inflammatory effect results from this mechanism, therefore having anti-cancer effects [1].

Since ω-3 PUFAs and ω-6 PUFAs compete for the same enzymes to produce oxylipins, increased ω-3 PUFAs decrease the production of ω-6-derived oxylipins like PGE2 and act as a natural COX inhibitor, inhibiting tumor cell growth [19].

In order for the metastatic cell migration to occur, an upregulation of chemokine receptors is required. These chemokine receptors act as sensors for cell trafficking. A metabolite of DHA, resolvin D1 (RvD1), has been shown to have the ability to reduce the surface expression of CXCR4, a chemokine receptor crucial in chemotherapy, and CXCR4-mediated cell migration [1].

Finally, supplementation with ω-3 PUFAs has also been shown to be associated with better tolerability of chemotherapy for certain groups [62].

## 7. Omega-3 and Omega-6 and the Market: A Case Study in Portugal

### 7.1. Medicines and Natural Supplements Using ω-3 and ω-6

There is a wide range of food supplements available that are based on ω-3 and ω-6 fatty acids, with a particularly large variety focused on ω-3. Numerous brands offer different omega supplementation products, varying in source, formulation, and concentration. Some of the claims are reducing the CV risk and cholesterol levels and improving brain function in adults, while some are specific for children in order for them to have a better brain function and development, maintain eyesight, and, in the case of children diagnosed with Attention Deficit Hyperactivity Disorder (ADHD), to be used as a complement to the therapy used. There are also brands that offer supplements containing ω-6 in conjunction with ω-3, and some also include ω-9 (which is not a target subject of this review). These products are directed towards good CV health, skin health, immune function, and general well-being [63].

When it comes to medicines based on omega FAs, in Portugal, there is only one that has been authorized and commercialized since 2003. It is a medicine composed of ω-3 FAs in the EE form, having a total dose of 840 mg per soft gel capsule (460 mg of EPA and 380 mg of DHA). The therapeutic indication of this medicine is as a treatment of hypertriglyceridemia when diet alterations are not enough. It can be used as treatment to stage IV hypertriglyceridemia as a monotherapy and stage IIb/III hypertriglyceridemia in association with statins. In terms of posology, it is recommended to use two soft gel capsules per day, with the possibility to increase the dosage to four soft gel capsules if needed [64,65].

### 7.2. Market Perspectives and Legislation

ω-3 PUFAs supplements are the most prescribed food supplements, which justifies their large availability in the market. The market is also predicted to grow by 6.5% from 2023 to 2032, exceeding USD 4.2 billion by the year 2032 [44].

The legislation and entities regulating food supplements are generally different from those for medicines. This regulatory gap raises several concerns, particularly regarding quality control and product safety.

Unlike medicines, food supplements are not subject to mandatory pre-market testing to verify the accuracy of label claims, such as the exact dosage of active ingredients. As a result, many supplements may not contain the stated concentrations or may include unintended substances originating from the raw materials used in their production. For instance, ω-3 fatty acid supplements derived from fish oil can sometimes contain contaminants like methylmercury—a toxic compound known to pose serious health risks. Moreover, multiple studies have shown that ω-3 (and ω-6) fatty acid supplements are highly prone to oxidation during storage, with retail products frequently presenting significant levels of oxidation by the time they reach consumers, which may further reduce their efficacy and safety [66,67,68,69,70]. The absence of strict, harmonized testing requirements across markets allows such risks to persist, potentially compromising both consumer safety and the reliability of the health claims made by manufacturers.

In the European Union (EU), food supplements are regulated under the Food Supplements Directive 2002/46/EC [71], which establishes a harmonized framework for labeling, permitted vitamins and minerals, and the conditions under which they can be marketed across all member states. Oversight is provided by the European Food Safety Authority (EFSA) [3], which issues scientific opinions on the safety of ingredients and evaluates health claims under the Nutrition and Health Claims Regulation (EC) No 1924/2006 [72]. However, enforcement and market surveillance are primarily the responsibility of each member state, which can result in variable application of controls across the EU. This means that, although EFSA provides scientific guidance, the rigor of inspections and compliance monitoring may differ between countries.

In the United States, food supplements are controlled by the Food and Drug Administration (FDA) under the Dietary Supplement Health and Education Act (DSHEA, 1994) [73]. Under this framework, dietary supplements are regulated more like foods than medicines, and manufacturers do not need to obtain pre-market approval before marketing their products. Instead, the responsibility for ensuring safety and accuracy of labeling lies primarily with the manufacturer. The FDA intervenes post-market if a product is found to be unsafe or mislabeled. One key limitation of this system might be the lack of systematic pre-market evaluation of purity, potency, or efficacy, which can result in significant variability in product quality.

In Canada, food supplements are regulated more stringently, being classified as Natural Health Products (NHPs) [74] under the Natural Health Products Regulations (2004). These regulations require pre-market licensing, submission of safety and efficacy data, and compliance with strict labeling standards. Health Canada issues a Natural Product Number (NPN) or Homeopathic Medicine Number (DIN-HM) for approved products, which must be displayed on the label. This approach provides a higher degree of assurance regarding both product quality and consumer safety compared with the U.S. model.

Other regions, such as Australia, regulate supplements through the Therapeutic Goods Administration (TGA) [75], which requires complementary medicines to be listed or registered on the Australian Register of Therapeutic Goods (ARTG). The level of pre-market assessment depends on the product risk category, but even lower-risk supplements must meet manufacturing and labeling standards. In contrast, many low- and middle-income countries still lack robust regulatory frameworks, which may increase the risk of substandard or contaminated products entering the market.

Taken together, these examples highlight the diversity of regulatory approaches worldwide. Those differences underscore the need for international harmonization of standards to ensure consistent product quality, safety, and transparency across global markets.

### 7.3. Drug Interactions with ω-3 and ω-6

Although ω-3 and ω-6 PUFAs provide recognized therapeutic benefits, their potential interactions with drugs require careful consideration. Clinical studies have shown that ω-3 fatty acids may increase bleeding time, creating additive risks when combined with anticoagulants (e.g., warfarin) or antiplatelet agents (e.g., aspirin, clopidogrel) [65]. Similarly, ω-3 supplementation can enhance the blood pressure-lowering effects of antihypertensive drugs, requiring monitoring to prevent hypotension. In dyslipidemic patients, ω-3 can act synergistically with statins and fibrates, potentially amplifying lipid-lowering effects. Additionally, interactions with immunosuppressants (e.g., cyclosporine, tacrolimus) and anti-inflammatory agents (e.g., corticosteroids, NSAIDs) may influence inflammatory and immune responses [76].

Given these possible interactions, careful clinical monitoring is essential. Specific indicators should include INR (International Normalized Ratio) in patients receiving anticoagulants, blood pressure monitoring in those on antihypertensive therapy, and lipid profiles in dyslipidemic patients. For patients on immunosuppressive regimens, close monitoring of inflammatory markers and drug levels may also be warranted. Individualized dose adjustments, combined with appropriate dietary counseling, are crucial to ensuring the safety and efficacy of combined therapies.

## 8. Adverse Effects of Misuse

When talking about adverse effects of misuse of ω-3 and ω-6 PUFAs, it covers both not getting enough quantities on the daily diet or through supplementation, leading to a deficiency of these nutrients in the organism, overconsumption of fish or supplements from fish oils that can lead to a consumption of high levels of methylmercury, and the known adverse effects related with the use of ω-3 FA medicine.

Although rarely described in humans, a deficiency in ω-3 and ω-6 PUFAs can lead to dermatitis, renal hypertension, mitochondrial activity disorder, CVDs, type 2 Diabetes, impaired brain development, arthritis, depression, and decreased body resistance to infection. In short, it leads to the opposite of the health benefits that proper intake of these nutrients has and can highly compromise one’s health [2].

Another noteworthy adverse effect, previously mentioned in this review, is the possible overexposure to methylmercury. Methylmercury, present in some fishing areas, contaminates the fish that are one of the main sources of ω-3 PUFAs, is neurotoxic and can be especially harmful for the development of the central nervous system of the fetus. Since the legislation does not impose mandatory safety testing and content evaluation for food supplements, this problem can be carried on to the food supplements based on fish oils, presenting itself as a problem worth mentioning and raising awareness about [12].

When it comes to ω-3-based medicine, the most common adverse effects that are expected when using it are: abdominal distention, abdominal pain, obstipation, diarrhea, dyspepsia, flatulence, burping, gastroesophageal reflux, nausea, and vomiting [65].

## 9. Conclusions and Future Perspectives

Through this review, the importance of ω-3 and ω-6 PUFAs for human health becomes unquestionable, having many important health benefits. Despite those benefits, in many diets, such as the western diet, the ω-6:ω-3 ratio is very unbalanced in favor of the ω-6 PUFAs, which is not ideal and shows the importance of raising awareness among the general population and the health professionals to promote a healthier ratio closer to the 1:1, which is considered most effective in providing maximum health benefits. This raise of awareness should also focus on the health benefits offered by these nutrients, encouraging the population to incorporate ω-3 and ω-6 PUFAs in the daily diet through both foods and supplements in a more informed and conscious manner.

It is also noteworthy that ω-3 fatty acid–based medicines have been available on the market for several decades and have demonstrated both safety and efficacy, particularly in managing hypertriglyceridemia and supporting CV health. However, statins remain the first-line therapy in most clinical guidelines for dyslipidemia and CV risk reduction. This preference is largely due to the extensive evidence supporting the effectiveness of statins. As a result, ω-3-based therapies are often considered adjunctive rather than primary treatment, despite their favorable safety profile and therapeutic potential.

Another relevant aspect is the current regulatory framework governing food supplements, which varies significantly between countries and differs from the more stringent systems applied to medicines. One of the key challenges identified is the limited mandatory oversight regarding the verification of nutrient content in supplements. This can lead to discrepancies between the declared and actual concentrations of active ingredients. For example, in fish oil-based ω-3 supplements, insufficient quality control may allow the presence of environmental contaminants such as methylmercury, which could pose health risks if not adequately managed.

Internationally, efforts are ongoing to harmonize standards and improve transparency in this sector, ensuring the application of standardized manufacturing practices and enhancing the overall quality, safety, and reliability of supplements marketed to the public.

Furthermore, integrating controlled extraction technologies could improve reproducibility and guarantee the consistency of composition in supplement formulations. Such advancements may support the broader use of food supplements as part of preventive healthcare strategies, aligned with global public health goals.

It is also important to raise awareness in the scientific community for the need of developing more and better studies in order to better understand the role and mechanisms behind all the health benefits of the ω-3 and ω-6 PUFAs, especially for ω-6 PUFAs since many of its health benefits are still not fully understood because there are studies that conclude both beneficial and harmful effects for this nutrient, making the need for more in-depth studies evident. Another important measure is the development of clear, universal, and evidence-based recommendations on the dosages that should be taken daily.

Another measure that could be taken in the future is the incorporation of more complete and detailed information about the amount of ω-3 and ω-6 present in each product, as well as a brief description of the main health benefits associated with its consumption and the recommended daily amounts that each person should consume. This measure could be used, not only for ω-3 and ω-6 but also for other important nutrients, thus improving the ability of the consumer to make an informed and conscious decision and the general public’s literacy.

## Figures and Tables

**Table 1:** Content of ω-3 and ω-6 fatty acids in common foods. Values are expressed in grams per 100 g of edible portion. ω-3: Omega-3; ω-6: Omega-6; ALA: ɑ-Linoleic Acid; EPA: Eicosapentaenoic Acid; DHA: Docosahexaenoic Acid; LA: Linoleic Acid; ARA: Arachidonic Acid. Scientific names of the foods are provided in parentheses. Data were obtained from the indicated references.

Sources		ω-3			ω-6		References
		ALA (g/100 g)	EPA (g/100 g)	DHA (g/100 g)	LA (g/100 g)	ARA (g/100 g)	
Oil	Corn (*Zea mays*)	0.6	-	-	49.83	-	USDA FDC SR Legacy, Corn oil (FDC ID: 170375) [28]
	Sunflower (*Helianthus annuus*)	0.33	-	-	49.89	-	USDA FDC SR Legacy, Sunflower oil, linoleic (FDC ID: 170562) [28]
	Soybean (*Glycine max*)	7.6	-	-	51.36	-	USDA FDC SR Legacy, Soybean oil (FDC ID: 170782) [28]
	Canola (*Brassica napus*)	9.15	-	-	18.65	-	USDA FDC SR Legacy, Canola oil (FDC ID: 171007) [28]
	Safflower (*Carthamus tinctorius*)	0.1	-	-	12.72	-	USDA FDC SR Legacy, Safflower oil, linoleic (FDC ID: 170611) [28]
	Flaxseed (*Linum usitatissimum*)	53.37	-	-	14.33	-	USDA FDC SR Legacy, Flaxseed oil (FDC ID: 170564) [28]
Fish Oil	Salmon (*Salmo salar*)	-	13.3	18.23	-	-	USDA SR28, Fish oil, salmon (converted to g/100 g) [28]
	Sardine (*Sardina pilchardus*)	-	10.15	10.66	-	-	USDA SR28, Fish oil, sardine (converted to g/100 g) [28]
	Herring (*Clupea harengus*)	-	6.28	4.21	-	-	USDA SR28, Fish oil, herring (converted to g/100 g) [28]
Vegetables	Lettuce (*Lactuca sativa*), Raw	0.15	-	-	0.06	-	USDA FDC, Romaine lettuce, raw (FDC ID: 1102666) [28]
	Green Broccoli (*Brassica oleracea* var. *italica*), Raw	0.11	-	-	0.03	-	USDA FDC, Broccoli, raw (FDC ID: 11090) [28]
	Brussels Sprout (*Brassica oleracea* var. *gemmifera*), Raw	0.17	-	-	0.08	-	USDA FDC, Brussels sprouts, raw (FDC ID: 11098) [28]
Fish	Salmon (*Salmo salar*), Raw	0.09	0.89	1.19	0.15	0.05	USDA FDC, Atlantic salmon, raw (FDC ID: 15076) [28,29]
	Herring (*Clupea harengus*), Raw	0.19	1.09	1.01	0.22	0.1	USDA FDC, Herring, Atlantic, raw (FDC ID: 15039) [28]
	Sardine (*Sardina pilchardus*), Raw	-	0.51	1.16	0.06	0.04	USDA FDC, Sardine, canned in oil, drained (FDC ID: 15090) [28]
	Trout (*Oncorhynchus mykiss*)*,* Raw	0.1	0.15	0.5	0.37	0.05	USDA FDC, Rainbow trout, farmed, raw (FDC ID: 15114) [28]
	Cod (*Gadus morhua*)*,* Dried	-	0.02	0.62	0.03	0.12	USDA FDC, Cod, Atlantic, dried/salted (FDC ID: 15015) [28]
Seeds	Chia (*Salvia hispanica*), Dried	17.83	-	-	5.84	-	USDA FDC, Chia seeds, dried (FDC ID: 12006) [28]
	Walnuts (*Juglans regia*), Dried	6.64	-	-	34.02	-	USDA FDC, Walnuts, English, dried (FDC ID: 12155) [28,30]
	Hazelnuts (*Corylus avellana*), Dried	0.11	-	-	5.09	-	USDA FDC, Hazelnuts, dried (FDC ID: 12120) [28]
	Almond (*Prunus dulcis*), Dried	0.3	-	-	10.54	-	USDA FDC, Almonds, dried (FDC ID: 12061) [28]

**Table 2:** Statistics from many studies on the effect of different outcomes, adapted from [40]. RR: Relative Risk; CI: Confidence Interval; CVD: Cardiovascular Disease; MI: Myocardial Infarction; CHD: Coronary Heart Disease; n: number of participants.

Outcome	Studies	Pooled RR (95% Cl)	Heterogeneity
CVD Events	39 (n = 134,843)	0.95 (0.90–1.00)	41% (Moderate)
MI	24 (n = 130,487)	0.87 (0.80–0.96)	28% (Low)
CHD Events	28 (n = 131,306)	0.90 (0.84–0.97)	40% (Moderate)
Fatal MI	14 (n = 78,981)	0.65 (0.46–0.91)	29% (Low)
CHD Mortality	22 (n = 122,231)	0.91 (0.85–0.98)	29% (Low)

## Data Availability

No new data were created or analyzed in this study. Dare sharing is not applicable.

## Abbreviations

The following abbreviations are used in this manuscript:

ω-3	Omega-3 Fatty Acids
ω-6	Omega-6 Fatty Acids
ACC	Acetyl-CoA Carboxylase
AD	Alzheimer’s Disease
ADHD	Attention Deficit Hyperactivity Disorder
AEA	Anandamide
AHA	American Heart Association
ALA	ɑ-Linoleic Acid
APP	Amyloid Precursor Protein
ARA	Arachidonic Acid
ASAE	Autoridade de Segurança Alimentar e Económica
BBB	Blood-Brain Barrier
CAT1	Carnitine Acetyltransferase 1
CE	Cholesterol Esters
CHD	Coronary Heart Disease
CI	Confidence Interval
Cmax	Maximum Concentration
COX	Cyclooxygenase
cPLA2	Cytosolic Phospholipase A2
CRP	C-Reactive Protein
CV	Cardiovascular
CVD	Cardiovascular Disease
CYP	Cytochrome P450
DAG	Diacylglycerols
DGAV	Direção-Geral da Alimentação e Veterinária
DGLA	Dihomo-γ-Linoleic Acid
DHA	Docosahexaenoic Acid
DiHOME	Dihydroxy-Octadecenoic Acid
DPA	Docosapentaenoic Acid
EE	Ethyl Esters
EET	Eicosatrienoic Acid
EFSA	European Food Safety Authority
ELOVL	Elongase
EPA	Eicosapentaenoic Acid
ETA	Eicosatetraenoic Acid
FA	Fatty Acid
FADS	Fatty Acid Desaturase
FAO	Food and Agriculture Organization
FDA	Food and Drug Administration
FFA	Free Fatty Acid
GDNF	Glial-Derived Neurotrophic Factor
GLA	γ-Linoleic Acid
HDL	High-Density Lipoprotein
HF	Heart Failure
HFrEF	Heart Failure Reduced Ejection Fraction
HODE	Hydroxy-Octadecadienoic Acid
HPA	Hypothalamic-Pituitary-Adrenal
INFARMED	Autoridade Nacional do Medicamento e Produtos de Saúde, I.P.
IL	Interleukin
IsoPs	Isoprostanes
IU	International Units
LA	Linoleic Acid
LCFA	Long-Chain Fatty Acid
LDL	Low-Density Lipoprotein
LOX	Lipoxygenase
LPL	Lipoprotein-Lipase
LT	Leukotrienes
MACE	Major Adverse Cardiovascular Events
MCFA	Medium-Chain Fatty Acid
MI	Myocardial Infarction
mtNOS	Mitochondrial Nitric Oxide Synthase
NeuroPs	Neuroprostanes
NNT	Number Needed to Treat
NO	Nitric Oxide
PD	Parkinson’s Disease
PFC	Prefrontal Cortex
PG	Prostaglandin
PGI	Prostacyclin
PhytoPs	Phytoprostanes
PL	Phospholipids
PPAR	Peroxisome Proliferator-Activated Receptor
PSEN1	Presenilin 1
PSEN2	Presenilin 2
PUFA	Polyunsaturated Fatty Acid
ROS	Reactive Oxygen Species
RR	Relative Risk
RvD1	Resolvin D1
SCFA	Short Chain Fatty Acid
SDA	Stearidonic Acid
SPM	Specialized Pro-resolvin Mediator
SREBP	Sterol Regulatory Element-Binding Protein
TAG	Triacylglycerols
TGF	Transforming Growth Factor
TNF	Tumor Necrosis Factor
TXA	Thromboxane
USA	United States of America
VEP	Visual-Evoked Potential
VFA	Volatile Fatty Acid
VLCFA	Very Long-Chain Fatty Acid
VLDL	Very-Low-Density Lipoproteins
WHO	World Health Organization
